# Determination of Effect Sizes for Power Analysis for Microbiome Studies Using Large Microbiome Databases

**DOI:** 10.3390/genes14061239

**Published:** 2023-06-09

**Authors:** Gibraan Rahman, Daniel McDonald, Antonio Gonzalez, Yoshiki Vázquez-Baeza, Lingjing Jiang, Climent Casals-Pascual, Daniel Hakim, Amanda Hazel Dilmore, Brent Nowinski, Shyamal Peddada, Rob Knight

**Affiliations:** 1 Department of Pediatrics, School of Medicine, University of California, San Diego, CA 92093, USA; 2 Bioinformatics and Systems Biology Program, University of California, San Diego, CA 92093, USA; 3 BiomeSense Inc., Chicago, IL 60615, USA; 4 Janssen Research & Development, Spring House, PA 19002, USA; 5 Department of Microbiology, Centre de Diagnòstic Biomèdic (CDB), Hospital Clinic, University of Barcelona, 08036 Barcelona, Spain; 6 Biomedical Sciences Program, University of California San Diego, La Jolla, CA 92093, USA; 7 Center for Microbiome Innovation, Jacobs School of Engineering, University of California San Diego, La Jolla, CA 92093, USA; 8 Biostatistics and Computational Biology Branch, National Institute of Environmental Health Sciences (NIEHS), The National Institute for Health (NIH), Research Triangle Park, Durham, NC 27709, USA; 9 Department of Computer Science and Engineering, University of California San Diego, La Jolla, CA 92093, USA; 10 Department of Bioengineering, University of California San Diego, La Jolla, CA 92093, USA

**Keywords:** bioinformatics, microbiome, statistics, effect size

## Abstract

Herein, we present a tool called Evident that can be used for deriving effect sizes for a broad spectrum of metadata variables, such as mode of birth, antibiotics, socioeconomics, etc., to provide power calculations for a new study. Evident can be used to mine existing databases of large microbiome studies (such as the American Gut Project, FINRISK, and TEDDY) to analyze the effect sizes for planning future microbiome studies via power analysis. For each metavariable, the Evident software is flexible to compute effect sizes for many commonly used measures of microbiome analyses, including α diversity, β diversity, and log-ratio analysis. In this work, we describe why effect size and power analysis are necessary for computational microbiome analysis and show how Evident can help researchers perform these procedures. Additionally, we describe how Evident is easy for researchers to use and provide an example of efficient analyses using a dataset of thousands of samples and dozens of metadata categories.

## 1. Introduction

Power analysis for a univariate (or multivariate) outcome variable is not new. Numerous statistical packages are available (e.g., SAS) for a variety of experimental designs and outcome variables. For a given level of significance, a common challenge with any power analysis is the understanding of the underlying variability in the data and the value of the parameter of interest in the alternative hypothesis. Once the statistical parameter of interest is identified, researchers often conduct a pilot study to estimate mean differences and standard deviations and use these values, termed effect sizes [1,2,3], as the basis for conducting power analysis, i.e., sample size calculations for the larger study proposed in their research program. The larger the effect size, the stronger the statistical difference, and the fewer samples are needed for high statistical power.

This type of power analysis is important because of the limited resources available for experimental designs. Ensuring that researchers do not spend more resources than required to achieve a given statistical power is paramount. The problem is more complicated when it comes to microbiome studies because there are a variety of parameters one can base their designs on. Almost all parameters of interest, such as measures of α or β diversity are (nonlinear) functions of relative abundances of various taxa. Estimation of relative abundances using small pilot studies (i.e., N < 100) is not always satisfactory because the observed count data contain a large number of zeros. The preliminary estimates from a pilot study are potentially subject to large bias and uncertainties. Consequently, the determination of the effect size for a given parameter, say α diversity defined by Shannon’s entropy, is a difficult task. This article takes the first step towards addressing this challenging problem by making use of the recently created large databases such as the American Gut Project [4], TEDDY [5], and FINRISK [6]. These are very rich databases that continue to grow. They contain microbiome data on several thousands of individuals along with hundreds of commonly measured metadata and thousands of represented taxa. For each variable in the metadata, say, mode of birth, the user-friendly software Evident derives the effect size for a parameter of a researcher’s interest, such as Shannon’s entropy. Using this parameter, a researcher can then conduct a simulation study to derive power functions for different sample sizes [7].

Since microbiome datasets such as AGP, TEDDY, and FINRISK are very large and contain a large number of metavariables, we expect Evident to be a useful tool for deriving effect sizes for variables of common interest. Importantly, Evident takes user-inputted study data for the generation of results, so researchers can customize their analyses as they see fit. As new databases get constructed, Evident will access those to derive better and more refined effect size estimates that will be useful for planning microbiome studies. Evident is available both as a standalone Python package as well as a QIIME 2 plugin [8]. Currently, effect size analysis and power analysis for microbiome science can be performed using programming languages such as Python and R. However, these approaches are not designed for use with many metavariables. As a result, researchers must write custom code to iterate through the full dataset. With Evident, researchers can seamlessly explore the effect size of community differences in dozens of metadata columns at once and easily perform power analysis. The interactive component of Evident additionally makes this process easy to use and share. This scalability, flexibility, user-friendliness, and integration with existing microbiome software make Evident easier to slot into existing microbiome workflows over existing methods.

## 2. Materials and Methods

**Figure 1:**
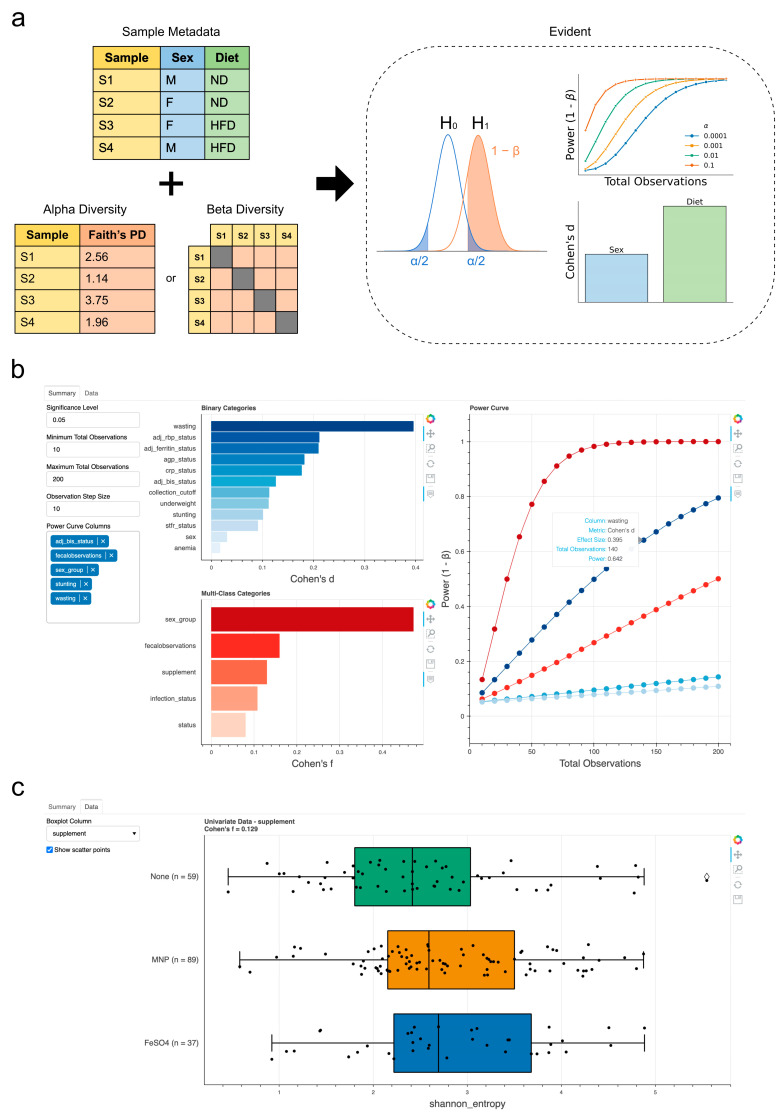
Evident workflow and interactive visualizations. (**a**) Graphical overview of Evident usage. Sample metadata with categorical groups are used to determine differences among samples. Effect size calculation can be performed and used to generate power curves (in this example using classification status from [7]) at multiple statistical significance levels and sample sizes. (**b**,**c**) Screenshots of the interactive webpage for a dynamic exploration of effect sizes and power analysis. Summarized effect sizes of all columns can be used to inform interactive power analysis on multiple groups (**b**). The underlying grouped data can be visualized with boxplots and, optionally, the raw data as scatter plots (**c**). The data shown are from McClorry et al. (Qiita study ID: 11402) [9].

Figure 1a shows an overview of the Evident workflow. As input, Evident takes a sample metadata file and a data file of interest (for example, α diversity). In this cartoon example [9], we show the main Evident workflow is (1) calculating effect size for a metavariable of interest between two or more groups (2) performing parametric power analysis on varying sample sizes, levels of significance, and/or effect sizes (3) plotting the accompanying power curve(s). Both univariate per-sample data (such as α diversity) and multivariate data (as a distance matrix such as β diversity) are supported. For univariate measures, the differences in means among groups are considered. For multivariate measures, the difference in means among within-group pairwise distances is considered. We also note that, at the moment, Evident implements effect size computations of univariable analyses (without explicit handling of confounders) following the approach of existing work [10,11,12,13].

Evident supports both binary categories and multi-class categories. For binary categories, Cohen’s d is calculated between the two levels. For multi-class categories, Cohen’s f is calculated among the levels [14]. Users also have the option of performing pairwise effect size calculations between levels of a multi-class category rather than comparing all groups together. Effect size calculations can be performed on multiple categories at once with simple parallelization by providing the number of CPUs to use. For example, this architecture allows us to decrease the runtime of effect size calculations for 9495 samples comprising 61 categories from over 12 min to 3.5 min using 8 CPUs in parallel.

Evident also provides an interactive component by which users can dynamically explore sample groupings. In Figure 1b,c, we show a screenshot of a web app that users can access with Evident. Metadata categories are pre-sorted by effect size, allowing efficient determination of interesting categories. Power analysis is implemented dynamically—multiple categories can be visualized simultaneously for a specified significance level and number of observations. Researchers can look at the “elbow” of the power curves to determine an optimal number of samples to achieve the desired statistical power for their experiments.

### 2.1. Statistical Methodology

Let $X_{1},X_{2},\ldots ,\;X_{l}$ denote *l* metavariables available in some database. Without loss of generality, in the following, we shall describe the methodology used in Evident for $X_{1}$. For simplicity of exposition, we shall drop the subscript 1 from $X_{1}.$ Furthermore, to fix ideas of the methodology and simplicity of exposition, we shall assume X is binary, such as mode of delivery. The outcome variable is denoted by Y, such as Shannon entropy, a measure of α diversity of an infant’s gut microbiome. The relative abundance of the $j^{th}$ taxon, j=1, 2 , …, q, in the $k^{th}$ infant belonging to the $X=i^{th}$ group, i=1, 2, …, G, (e.g., mode of delivery), is denoted by $p_{ijk}.$ For example, X=1 represents babies born vaginally, and X=2 represents babies born by C-Section. We assume that there are q taxa measured on each infant (some may be zeros) and there are $N_{i}$ infants in the $i^{th}$ the group in the large database. Thus, the Shannon entropy for the $k^{th}$ subject belonging to the $X=i^{th}$ group is given by $Y_{ik}=-\overset{q}{\underset{j=1}{\sum }}p_{ijk}\ln\left(p_{ijk}\right)$. In this definition, $p_{ijk}\ln\left(p_{ijk}\right)\to 0,\;\mathrm{as}\;p_{ijk}\to 0$. Since we are working with very large databases, such as AGP, we assume that each $N_{i}$ is sufficiently large.

The Evident methodology for determining the effect size needed for conducting power analysis and sample size calculations for a future infant gut microbiome study using Shannon entropy to describe microbial diversity is described in the following steps.

Step 1 (Average population diversity): For each value of X, for each subject in the database, using the available microbiome data, compute the desired parameter of interest, for example, the average Shannon entropy for α diversity, $\mu_{i}=-\left(\frac{1}{N_{i}}\right)\overset{N_{i}}{\underset{k=1}{\sum }}Y_{ik},\;i=1,\;2$. As noted above, we assume that each $N_{i}$ is sufficiently large so that $\mu_{i}$ represents the average Shannon entropy, for the $i^{th}$ the population of infants.

Step 2 (Variance of population diversity): Similar to the population mean $\mu_{i},$ for each i=1, 2, …G we compute the population variance of α diversity, denoted by $\sigma_{i}^{2}=\left(\frac{1}{N_{i}}\right)\sum_{k=1}^{N_{i}}{\left(Y_{ik}-\mu_{i}\right)}^{2}\;.$ Again, each $n_{i}$ is sufficiently large so that $\sigma_{i}^{2}\;$ represents the population variance of Shannon entropy for the $i^{th}\;$ population of infants. Under the simplifying assumption of homoscedasticity (i.e., all populations have same the variance), we average the two empirical variances to obtain the pooled variance, i.e., $\sigma_{pool}^{2}=\frac{\sum_{i=1}^{G}N_{i}\sigma_{i}^{2}}{\sum_{i=1}^{G}N_{i}}$. Again, since the sample sizes are large, we regard the pooled variance as the true population variance for all our calculations.

Step 3 (Effect size calculations): Assuming that the outcome variable of interest (e.g., α diversity) is normally distributed, we have the following formulas for effect sizes using non-central distribution for the test statistic (for G=2) or non-central F distribution (for G≥2), respectively:

$$d=\frac{\mu_{1}-\mu_{2}}{\sigma_{pool}}\;.$$

$$f=\frac{\sum_{i=1}^{G}\left(\frac{N_{i}}{N}\right){\left(\mu_{i}-\bar{\mu }\right)}^{2}}{\sigma_{pool}^{2}}\;,\;\bar{\mu }=\sum_{i=1}^{G}\frac{N_{i}}{N}\mu_{i},\;N=\sum_{i=1}^{G}N_{i}.$$

Although equal variances across groups may be an unreasonable assumption, in the following we make a simplifying assumption that all groups have a common variance of $\sigma_{pool}^{2}$.

Step 4 (Power and sample size calculations): For a future study, suppose a researcher has a budget for a sample size $m_{i}$, for the $i^{th}\;$ population of infants, i=1, 2, …, G, then for a level of significance of α, the power corresponding to the effect size d and sample $m_{i}$, can be calculated parametrically, assuming $Y_{ik}\;\sim^{iid}\;N\left(\mu_{i},\sigma_{pool}\right)$.

Under the normality assumption, for G=2, Evident calculates power using non-central t-distribution using the effect size parameter d and different choices of samples sizes $m_{1},\;m_{2}$. In the case G>2, it uses non-central F distribution with effect size parameter f and different choices of samples sizes $m_{1},\;m_{2},\;\ldots ,\;m_{G}$.

### 2.2. Interactive Exploration of Community Differences

The interactive visualization provided in Evident is created with Bokeh. Given microbiome data and sample metadata, Evident creates a Bokeh app that dynamically calculates effect sizes and power analysis for the chosen parameters. This view also shows the raw data values as boxplots with optional scatter points.

### 2.3. Analysis of AGP Data

A sample ID list was generated from the original distance matrix used in the AGP study. 100 nucleotide 16S rRNA gene amplicon (16S) data targeting the V4 hypervariable region for these samples were downloaded from the AGP study on Qiita (study ID: 10317) using redbiom [15,16]. Both preparation and sample metadata were also retrieved with redbiom. Due to multiple preparations containing data from some samples, we performed disambiguation by keeping the samples with the highest sequencing depth.

We then processed the feature table and metadata according to the original study. The original workflow used the default parameters in Deblur to remove features with fewer than 10 occurrences in the data [17]. Because Qiita does not perform this filtering by default, we performed this filtering manually. To remove sequences associated with sample bloom, we performed bloom filtering [18]. We then rarefied the feature table to 1250 sequences as in the original analysis.

We processed the sample metadata in accordance with the original study. Because of differences in self-reporting protocols from 2018, metadata categories associated with reported Vioscreen responses as well as those associated with alcohol consumption were removed. The following categories were removed due to mismatches in sample metadata: roommates, allergies, age_cat, bmi_cat, longitude, latitude, elevation, height_cm, collection_time, and center_project_name. Only the top four annotated countries were considered—US, UK, Australia, and Canada. All other countries were ignored. Overall, 61 metadata categories common to both the original data and redbiom data were used for further analysis.

Sequences from the feature table were placed into a 99% Greengenes [19] insertion reference tree using SEPP [20]. We then used unweighted UniFrac to generate a sample-by-sample distance matrix [21]. This distance matrix was used as input to Evident along with the disambiguated, processed sample metadata.

We used effect_size_by_category to calculate the whole-group effect sizes for each column in the metadata and pairwise_effect_size_by_category to calculate the group-pairwise effect sizes for multi-class categories. For each whole-group effect size, we computed a power analysis for α values of 0.01, 0.05, and 0.1. Power was calculated on total sample size values from 20 to 1500 in increments of 40 samples. Evident analyses were performed in parallel on a high-performance computing environment. Group-wise and pairwise effect size calculations both took under 4 min for 82 metadata categories on 9495 samples using 8 CPUs (we note the AGP paper used n = 9511 but operated at 125 nt; we observe a slightly reduced number of samples at 100 nt). We also benchmarked group-wise effect size calculations using only a single CPU as a comparison; this process took 12.4 min, meaning the parallelization decreased runtime by approximately 3.5×. Power analysis calculation took 2.7 min for 82 categories using 8 CPUs in parallel.

### 2.4. Analysis of Study of Latinos Data

We downloaded closed-reference (picked against Greengenes 97%) 16S-V4 fecal data from Qiita (study ID: 11666) using redbiom. We used the bmi_v2 column to separate samples into two groups: normal (BMI < 25) and obese (BMI > 40). For each sample, we summed the abundance of *Prevotella* spp. and *Bacteroides* spp. adding a pseudocount to both sums. We then calculated the (log) ratio of the *Prevotella* sum and *Bacteroides* sum.

For power analysis, we first established the “true” difference between the obese and normal samples as 1.06 (d = 0.27). We used the log-ratio data to determine three levels of effect sizes we wanted to evaluate: 0.5 (d = 0.13), 1.0 (d = 0.25), and 1.5 (d = 0.38). To convert the differences into desired effect sizes, we divided each difference by the pooled standard deviation of the original log ratios. We used Evident to compute the power at each of these effect sizes for a significance threshold of 0.05 for total observations varying between 100 and 1000.

## 3. Results

**Figure 2:**
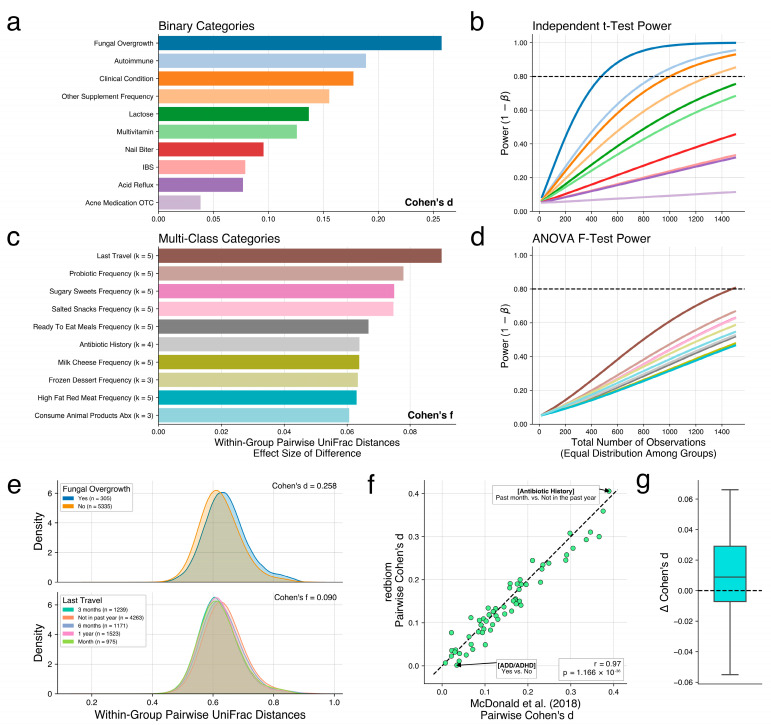
Analysis of American Gut Project data. (**a**) Top 10 binary categories by group-wise effect size. (**b**) Two-sample independent t-test power analysis of selected binary category effect sizes for a significance level of 0.05. (**c**) Top 10 multi-class categories by group-wise effect size. (**d**) One-way ANOVA F-test power analysis of selected multi-class category effect sizes at a significance level of 0.05. (**e**) Distributions of within-group pairwise UniFrac distances for highest effect size binary category (top) and multi-class category (bottom). (**f**) Comparison of pairwise effect sizes between reprocessed data from redbiom and published effect sizes from McDonald et al. Reprocessing results are not identical due to inherent randomness in rarefaction. (**g**) Boxplot of differences in effect sizes between published and reprocessed effect sizes.

As a demonstration of Evident, we reprocessed 9495 samples from the AGP to compare the published effect sizes in McDonald 2018 with those from a new analysis with Evident [4]. We downloaded the same samples from the original paper and reprocessed the data and metadata in the same manner, focusing on within-group UniFrac [22] distances. First, we computed the group-wise effect sizes for all valid metadata categories. The top ten binary categories and multi-class effect sizes are shown in Figure 2a,c, respectively. Using these effect sizes, we performed power analyses for each category at a significance level of 0.05 for a range of sample sizes from 20 to 1500 (Figure 2b,d). We plotted the distribution of the highest effect size binary and multi-class categories as reported by our new analysis in Figure 2e. Finally, we computed the pairwise effect sizes as performed in the original paper to verify that Evident returns the same values. Figure 2f shows that the effect sizes map extremely closely between the published data and the newly reprocessed data. The values of effect size differences in Figure 2g are distributed around 0, indicating that there is very little difference between effect size calculations. This serves as validation that Evident returns the correct effect sizes. We note, however, that the data used in this study is very heterogeneous—coming from multiple countries. It is important to make sure the data used in computing effect sizes are specific to the biological questions of interest. In Supplementary Figure S1, we plot the effect sizes calculated from only US samples and only UK samples. These effect sizes have a weak correlation (Spearman rho = 0.54), suggesting that country is a strong factor for effect sizes between biological groups. As such, researchers may want to perform further pre-processing such as stratification of data by country and computing the individual effect sizes for each population. We believe more work should be done on evaluating these differences in relation to these heterogenous populations to ensure results are not artificially inflated or deflated.

While we focus on diversity measures in this work, Evident is also usable with any other data such as log ratios of microbial abundances. As an example, we use Evident to extend the work of Morton et al. [23] and Fedarko et al. [24] in using log ratios for, e.g., post-hoc differential abundance analysis. We analyzed the commonly reported (log) ratio of *Prevotella* to *Bacteroides* in the Study of Latinos (SoL) cohort [25]. In Supplementary Figure S2, we plot the log ratio differences between subjects with a BMI < 25 and subjects with a BMI > 40. We also plot a power curve with custom differences in means, showing Evident’s flexibility in designing experiments with specific effect sizes in mind.

## 4. Conclusions

It is important for researchers to keep effect sizes in mind when performing computational microbiome analysis. Calculating and reporting effect sizes make it easier for researchers to determine the magnitude of biological effects on microbial communities. Additionally, these effect sizes can be used to inform power analyses for the efficient allocation of resources for new studies. We designed Evident for researchers to easily mine and process existing datasets for this information. Evident can slot into existing microbiome workflows and process numerous metadata categories efficiently and quickly, allowing its application to a broad range of microbiome research questions.

We note that the choice of study used in Evident should be carefully considered when designing and planning new experiments. For example, an existing study using 16S sequencing may not be completely appropriate when planning shotgun metagenomics experiments, or even experiments that will use a different primer set to target a different region of the 16S rRNA gene, because the different methods may recapture different bacteria with different efficiencies and therefore the effect size of the same per-subject or per-sample variable may differ depending on the methodology. More work should be done to evaluate the differences in downstream analyses on samples between 16S and shotgun metagenomics data. Similarly, culture-based microbiome studies may not follow the same statistical properties as NGS data. Researchers should be mindful of these differences when using Evident. Additionally, researchers should be aware of the limitations of the statistical methodology of Evident. For example, if the assumptions of variance homogeneity are not held, the obtained effect sizes will be inaccurate and the subsequent power analyses can overestimate or underestimate the number of samples required to achieve a given level of statistical power. Similarly, the assumption of equal group sizes in proposed experimental designs from power analysis may be naïve in practice. For rare diseases or phenotypes, it may not be feasible to design an experiment in which all groups have the same number of samples. In these cases, performing simulations with unequal group sizes to determine the necessary sample size to be likely to achieve a statistically significant result may be informative.

We encourage microbiome researchers to incorporate Evident into their workflows for both reporting effect sizes of microbial community differences and planning experimental designs. In the future, we hope to enhance flexibility by including quantitative metadata categories (rather than the current qualitative categories) and unbalanced group sample size power analyses.

## Data Availability

Data for the demonstration in Figure S1 were downloaded from Qiita (study ID: 11402) [9] at 90 nucleotides using the deblur pipeline. AGP data were downloaded from Qiita (study ID: 10317) using redbiom with context “Deblur_2021.09-Illumina-16S-V4-100nt-50b3a2”. The original pairwise effect sizes, sample metadata, and unweighted UniFrac distance matrix were downloaded from the original McDonald et al. study for comparison. SoL data used in Supplemental Figure S2 were downloaded from Qiita (study ID: 11666) using redbiom with context “Pick_closed-reference_OTUs-Greengenes-Illumina-16S-V4-90nt-44feac”.

## Supplementary Materials

The following supporting information can be downloaded at: https://www.mdpi.com/article/10.3390/genes14061239/s1, Figure S1: Comparison of effect sizes between UK and US samples; Figure S2: Log-ratio analysis on Study of Latinos cohort.

## Code Availability

The latest version of Evident is available at https://github.com/biocore/evident under the BSD-3 license. Evident is installable from PyPI both as a standalone Python 3 package and a QIIME 2 plugin. The scripts used to download and analyze AGP data as well as the processed Evident results are available at https://github.com/knightlab-analyses/evident-analyses. Analysis of AGP data in this study was performed with Evident version 0.4.0.
