# Supplementary material for: Determination of Effect Sizes for Power Analysis for Microbiome Studies Using Large Microbiome Databases
Source: Genes (Basel). 2023 Jun 9;14(6):1239. doi: 10.3390/genes14061239 (PMC10297957; doi:10.3390/genes14061239)
Supplement: Supplementary file 1 [file genes-14-01239-s001.zip › genes-2403416-supplementary.pdf]

Supplemental Figures

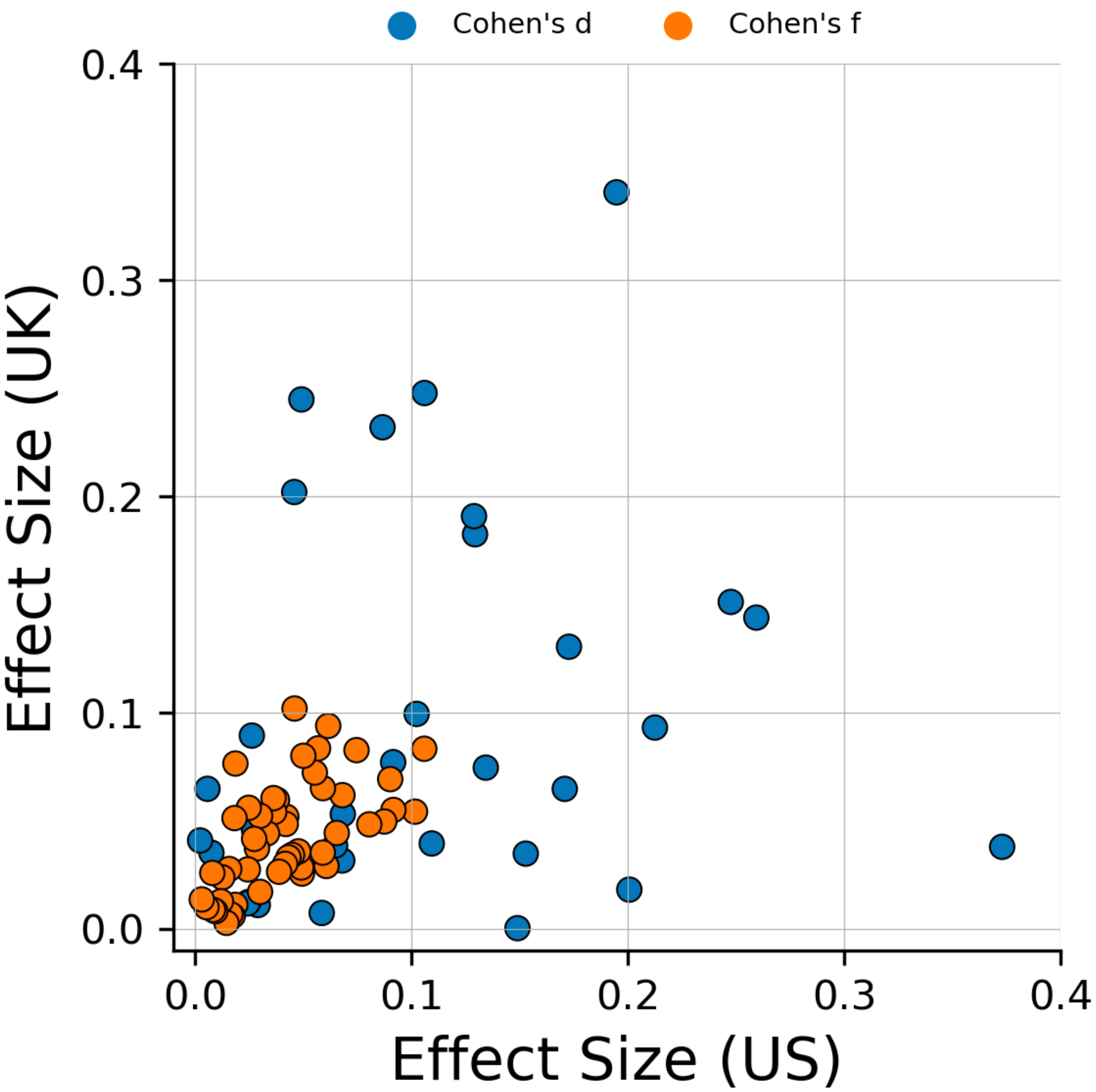

Supplementary Figure S1: Comparison of effect sizes between UK and US samples
Scatterplot showing comparison between effect sizes calculated from only UK samples and effect sizes calculated from only US samples (Spearman rho = 0.54).

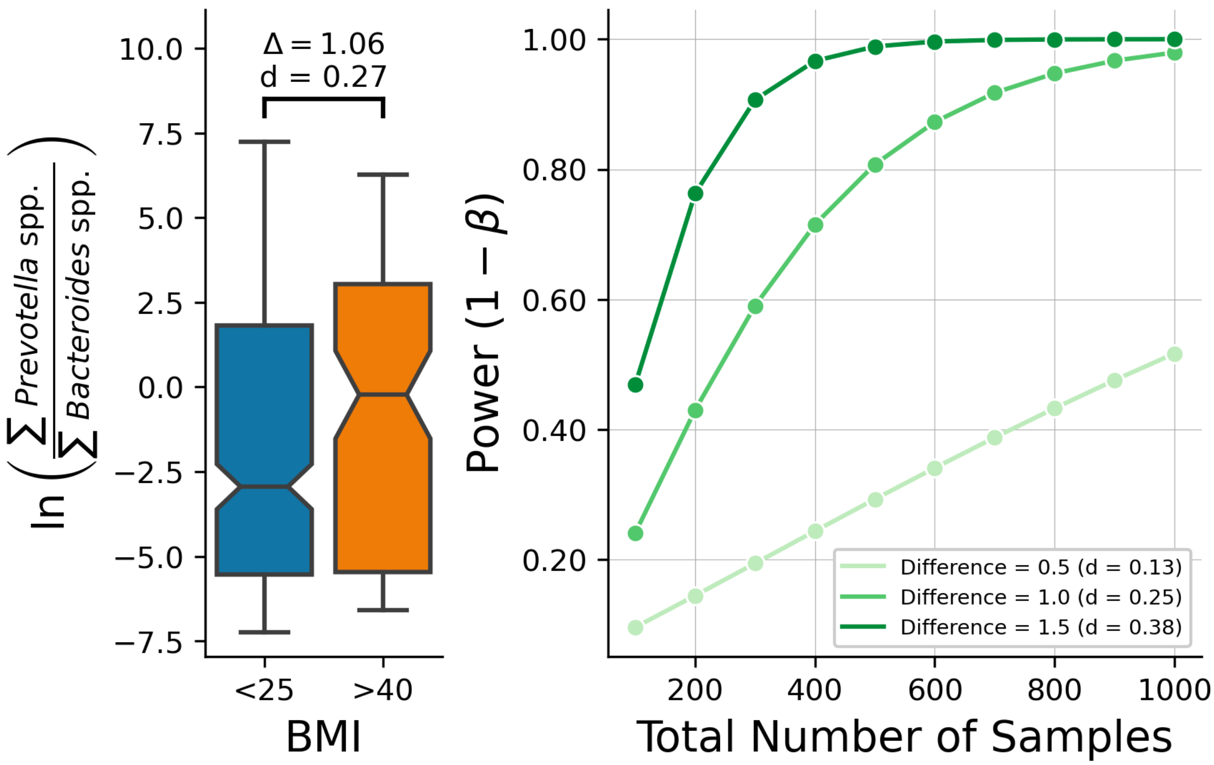

Supplementary Figure S2: Log-ratio analysis on Study of Latinos cohort
(Left) Boxplot showing comparison of *Prevotella*:*Bacteroides* log-ratios between subjects with BMI < 25 and subjects with BMI > 40. (Right) Power curve of log-ratio analysis at varying differences in means between groups corresponding to different effect sizes.
